# Toward Guidelines for Designing Holistic Integrated Information Visualizations for Time-Critical Contexts: Systematic Review

**DOI:** 10.2196/58088

**Published:** 2024-11-20

**Authors:** Ahmed Mohammed Patel, Weston Baxter, Talya Porat

**Affiliations:** 1 Dyson School of Design Engineering Imperial College London London United Kingdom

**Keywords:** visualization, design, holistic, integrated, time-critical, guidelines, pre-attentive processing, gestalt theory, situation awareness, decision-making, mobile phone

## Abstract

**Background:**

With the extensive volume of information from various and diverse data sources, it is essential to present information in a way that allows for quick understanding and interpretation. This is particularly crucial in health care, where timely insights into a patient’s condition can be lifesaving. Holistic visualizations that integrate multiple data variables into a single visual representation can enhance rapid situational awareness and support informed decision-making. However, despite the existence of numerous guidelines for different types of visualizations, this study reveals that there are currently no specific guidelines or principles for designing holistic integrated information visualizations that enable quick processing and comprehensive understanding of multidimensional data in time-critical contexts. Addressing this gap is essential for enhancing decision-making in time-critical scenarios across various domains, particularly in health care.

**Objective:**

This study aims to establish a theoretical foundation supporting the argument that holistic integrated visualizations are a distinct type of visualization for time-critical contexts and identify applicable design principles and guidelines that can be used to design for such cases.

**Methods:**

We systematically searched the literature for peer-reviewed research on visualization strategies, guidelines, and taxonomies. The literature selection followed the PRISMA (Preferred Reporting Items for Systematic Reviews and Meta-Analyses) guidelines. The search was conducted across 6 databases: ACM Digital Library, Google Scholar, IEEE Xplore, PubMed, Scopus, and Web of Science. The search was conducted up to August 2024 using the terms (“visualisations” OR “visualizations”) AND (“guidelines” OR “taxonomy” OR “taxonomies”), with studies restricted to the English language.

**Results:**

Of 936 papers, 46 (4.9%) were included in the final review. In total, 48% (22/46) related to providing a holistic understanding and overview of multidimensional data; 28% (13/46) focused on integrated presentation, that is, integrating or combining multidimensional data into a single visual representation; and 35% (16/46) pertained to time and designing for rapid information processing. In total, 65% (30/46) of the papers presented general information visualization or visual communication guidelines and principles. No specific guidelines or principles were found that addressed all the characteristics of holistic, integrated visualizations in time-critical contexts. A summary of the key guidelines and principles from the 46 papers was extracted, collated, and categorized into 60 guidelines that could aid in designing holistic integrated visualizations. These were grouped according to different characteristics identified in the systematic review (eg, gestalt principles, reduction, organization, abstraction, and task complexity) and further condensed into 5 main proposed guidelines.

**Conclusions:**

Holistic integrated information visualizations in time-critical domains are a unique use case requiring a unique set of design guidelines. Our proposed 5 main guidelines, derived from existing design theories and guidelines, can serve as a starting point to enable both holistic and rapid processing of information, facilitating better-informed decisions in time-critical contexts.

## Introduction

### Background

The need for quick and holistic processing and understanding of information or sensemaking is critical across multiple domains, particularly in health care. We define *quick* as ±30 seconds and *holistic* as representing *the bigger picture*, where all core components are referenced and individual components can only be understood in relation to the whole. A holistic visualization allows users to grasp the entire picture without needing to consult multiple sources. Examples include providing primary care practitioners with a holistic overview of the patient before starting a clinical consultation, with the requirement from clinicians to assimilate and understand the patient’s key information within 30 seconds [1], or a holistic display for antibiotic use in intensive care that can be perceived in <40 seconds [2].

Enhanced processing speed, where an individual can understand and react to the information received in a holistic manner, can enable rapid or real-time response and contribute to heightened situational awareness and informed decision-making [3]. With the vast amount of information available from multiple and heterogeneous sources of data, considered organization is required more than ever to guide both the understanding and interpretation of the information [4-6].

While numerous guidelines exist for a range of visualizations, such as designing visualizations more broadly [5,7-10], step-by-step processes for visualizing data [9,11-13], designing dashboards [14-16], and designing glanceable visualizations [17,18], guidelines toward enhancing situational awareness [19] and visualization architecture for time-critical contexts, as well as merging support for decision-making with situational awareness [20], this study evidences that there are currently no guidelines for the design of holistic integrated visualizations enabling quick information processing and comprehension in time-critical contexts.

In this paper, we argue that, for time-critical contexts, holistic visualizations that integrate multidata variables into a single visual representation are a distinct type of information visualization with different needs and constraints. We define *integrated* as a single connected visual representation of multidata variables (ie, different data dimensions). Data dimensions pertain to the categories that define the structure of the information (ie, medications or allergies in a health care visualization or altitude or speed in a military aircraft display) and represent the characteristics of the described data. Data points are the individual variables and data instances within the data dimensions (ie, allergy to penicillin). An integrated presentation brings together diverse data in a single visual representation, allowing users to view and analyze the data in a way that is interconnected, showing the links and relationships within the data (ie, between medication and allergies). Thus, holistic integrated visualizations combine both holistic and integrated approaches. While *holistic* references the core information, allowing users to grasp the entire picture (ie, that the patient is an older adult female, who is obese, smokes, and has uncontrolled diabetes and hypertension), *integrated* provides a single connected visual representation of these different data dimensions, allowing the user to understand the relationships between the patient’s characteristics (ie, between allergies and medications or health conditions and medications). As characteristics refer to the distinctive and discernible attributes, features, and qualities of something, the characteristics of holistic integrated information visualizations (HI-viz) are both holistic (offering the *bigger picture* and a comprehensive overview of key information) and integrated (a unified, connected visual representation), with higher information complexity (diverse data dimensions), and timely (enabling rapid processing of information in time-critical contexts). Toward defining design guidelines for this unique type of visualization, which we name HI-viz, we systematically searched the literature for established visualization design theories and guidelines from academic and industry sources, focusing on general design guidelines for visualizations and more specific guidelines for designing holistic, integrated, and timely visualizations.

In the following sections, we will position HI-viz displays against the current existing visualization types.

### Visualization Types

#### Glanceable Visualizations

*Glanceable visualizations* are typically discussed in the context of smartwatches or smartphones [18,21-23] and characterized as *“*being able to quickly extract the essential information from a display by a quick glance*”* [18,24]. Example scenarios include a runner who has a few hundred milliseconds to glance at the smartwatch to check relevant indicators, such as heart rate, elevation, and distance covered, before refocusing on the trail ahead [21,23]. The quick information needs support the shifting of focus from the primary task, typically in use cases that are locomotive, and through displays that are portable or small in the case of a smartwatch. The information provided is immediate, featuring a simple appearance and encoding very few data dimensions [23], be it in the form of a graph [18,23] or abstracted representations such as an arrow indicating direction or the use of other familiar visual metaphors [21,22]. Previous human-computer interaction studies investigating smartwatch use and interactions [25,26] have categorized timescales of smartwatch glanceable interactions as *peeks* (≤5 seconds) and *interactions* (>5 seconds). Up to 5 seconds can be taken as the limits of a glanceable visualization without any physical interactions, where the user receives, understands, and acts on the information. However, the time a user actively attends to the content via a “glance” or “peek” is suggested to be shorter [23].

#### Individual Visualizations

*Individual visualization types* such as line, bar, radial, and donut graphs are established visual representations in glanceable visualizations [18,23,27]. The effectiveness of these varies. Comparative studies highlight that information presented in radial charts takes longer to gather than that presented in bar or donut charts [23,28]. A study on information representation types in smartwatches [29] highlighted “icon+text” as being the most common representation type and supports research suggesting that textual data representations are predominant in smartwatches [30]. The most common graphical representation types or information “visualization techniques” are categorized as trees, scatter plots, charts, tables, diagrams, and graphs [31,32]. Previous studies have evaluated the effectiveness of different visualization types [33-38] as well as effectiveness across a number of selected tasks [33,38-41]. While the various visualization types perform differently depending on the task at hand, the literature suggests a positive correlation between perceived accuracy and user preference in 2D visualization types (tables, line charts, bar charts, scatterplots, and pie charts) for facilitating accurate and quick completion of tasks (finding anomalies, finding clusters, correlation, derived value, distribution, finding extremum, order, retrieving value, filtering, and determining range). Average response times reported in the study ranged between 10 and 40 seconds [33].

#### Increased Complexity and Visualization Types

A traditional individual visualization type typically encodes up to 3 data dimensions, this being the number of unique attributes or discrete information points in the dataset [42]. When more data dimensions are included, the *complexity* is increased. Commonly, 3 to 4 data dimensions are encoded in a visualization [43]. For example, a line graph representing an individual’s step count over the course of a week illustrates the days and number of steps—2 data dimensions. Adding a second individual to the graph provides a third data dimension. Adding data points within these dimensions (ie, additional weeks or more individuals) will not increase the complexity but may make it more crowded or complicated to visualize and understand the graph [43]. As the number of data dimensions that need to be visually encoded increases, the *complexity* of a visualization increases [43]. Although applications of visualizations encoding ≥4 data dimensions exist, it becomes more challenging to design and learn from them and illustrate their relationships [43,44]. Perceptually, an increase in dimensionality can also be overwhelming [42]. Beyond 3 data dimensions, visualization representations include *combined visualization types* (such as a line graph integrated within a bar graph); individual visualization types collectively tiled to present the information (*dashboards*); *diagrammatic representations* (ie, concept diagrams and scientific overviews) visualizing relationships between parameters, connecting and linking various themes to represent a multidimensional concept or topic [45,46]; or aesthetic presentations (*infographics* and *visual art*).

As the data dimensionality increases and visual representations change, the reader requires more time to understand the visualization [47,48]. There is also a shift from *explanatory visualizations*, where the data and narrative are provided, structured, and communicated, toward *exploratory visualizations*, where the viewer seeks the story that the data have to tell them through open interactions [43,49]. Iliinsky and Steele [43] further propose a hybrid explanation-exploration category where the provided data are curated and communicated, allowing for some exploration and interaction; thus, the reader can “...choose and constrain certain parameters, thereby discovering for herself whatever insights the dataset may have to offer.” Adopting a hybrid approach and finding a balance between explanation and exploration contributes to some of the most successful visualizations of today [49]. *Dashboards* are an example of the hybrid exploration-explanation approach, where multiple individual visualizations provide a top-level overview, encouraging further exploration and analysis [50]. The literature characterizes dashboards as being “glanceable” [50,51] because of the tiled layout of singular simple charts or numbers, which facilitates the information to be monitored at a glance [52]. However, studies evaluating efficiency or task completion rate via dashboards highlight the engagement and time for task completion to be several minutes [53-55].

*Infographics* are common in data journalism, with the goal of making information presentable and digestible to readers [56,57]. Defined as *“*a larger graphic design that combines data visualisations, illustrations, text, and images together into a format that tells a complete story” [58], infographics are highly engaging [56] and exploratory [43,51], intended for “deeper exposition” [50]. Infographics provide a high-level overview or essence of complex, abstract, and dense information [59,60], typically static when published [61] and rendered through a manual illustration process, consequently being aesthetically rich but making it challenging to change and manipulate data points [43]. Beyond infographics, the *visual art* use case is highly exploratory, and data are translated into a visual form typically with a “unidirectional encoding of information*”* [43]. The viewer is presented with a highly visual but abstract representation and seeks to interpret and arrive at their own meaning of the data [43,62].

Existing visualizations support both explanatory and exploratory tasks [43]. *Explanatory visualizations* typically encode 1 to 3 data dimensions [42], communicating information and data, including glanceable visualizations and scientific individual types (eg, line, bar, radial, and donut graphs). Visualizations combining individual types (line graph included within a bar chart) encode ≥4 data dimensions [43]. *Exploratory visualizations* are higher in data dimensionality, typically where the viewer interacts with the visualization to seek the story communicated by the data (eg, infographics and visual art) [43,44]. Some visualization types, including dashboards, diagrammatic representations, and some infographics and holistic integrated visualizations, can be described as being both explanatory and exploratory through a combination of information communication and structured interaction [43,49]. HI-viz is positioned at the forefront of the explanatory-exploratory Venn diagram due to the heightened complexity and required rapid processing time akin to that for explanatory visualizations.

**Figure 1:**
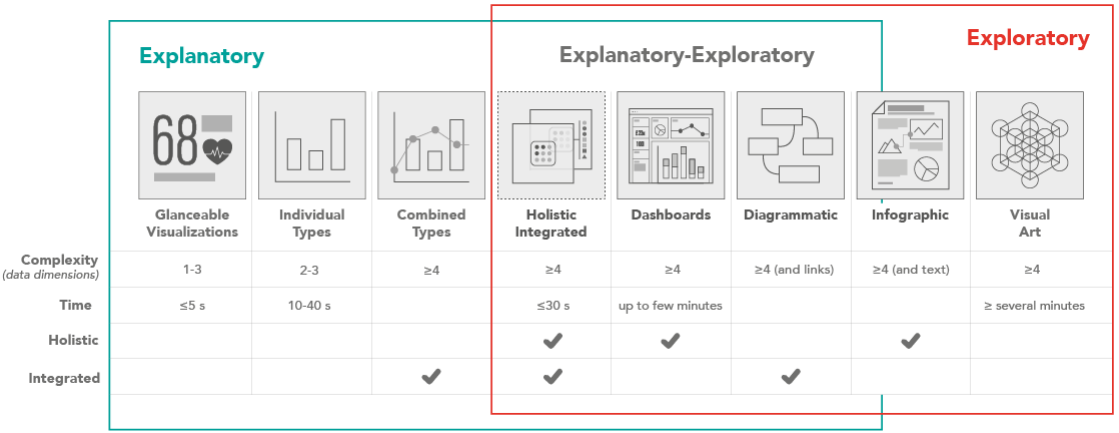
Characteristics of different visualization types—categorization by information complexity (number of data dimensions represented), time (for interpretation), and being holistic (overview of core information) and integrated (representation of multi-data variables in a single connected visualization.

**Figure 2:**
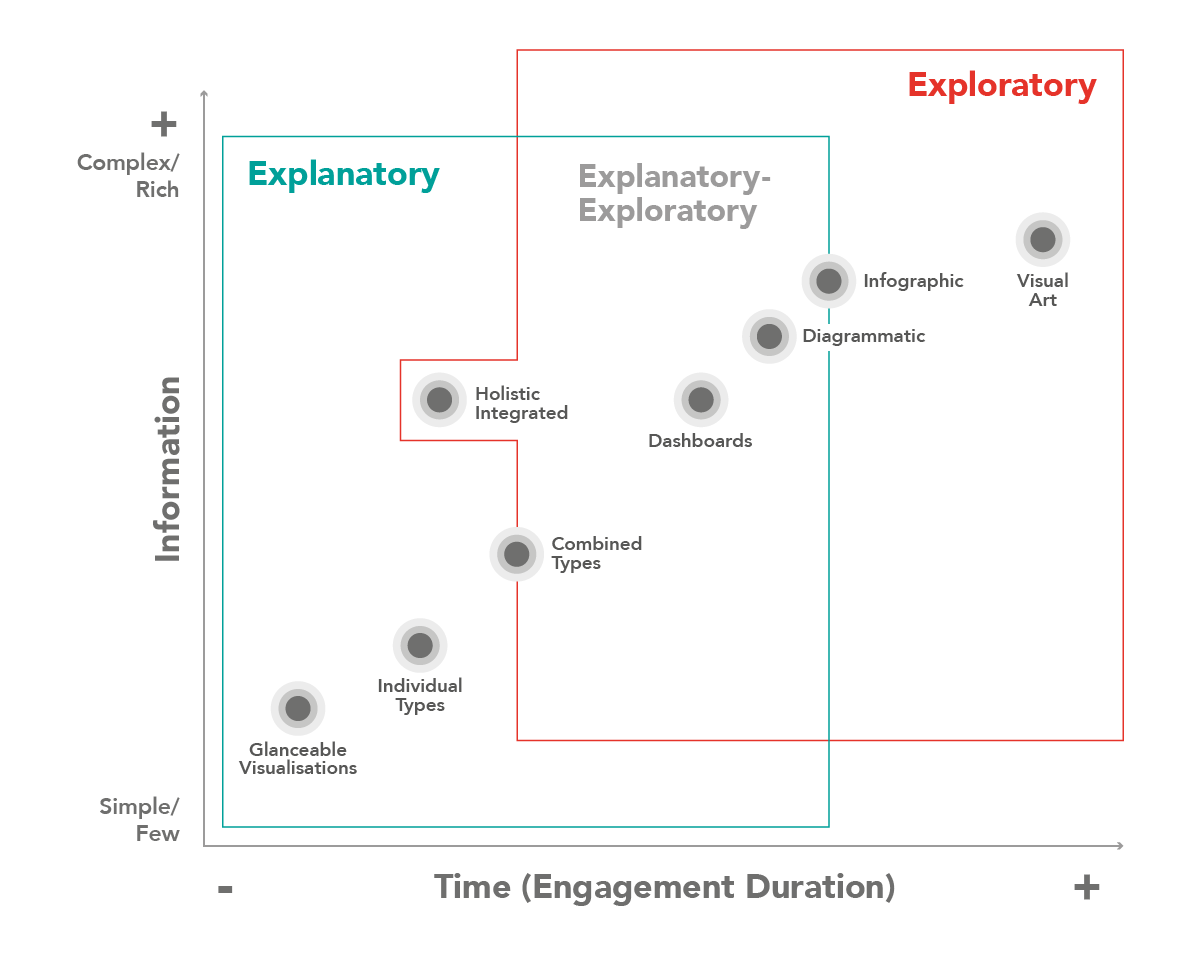
Positioning of holistic integrated information visualizations (HI-viz)—information visualization types plotted against information complexity (from simple visualizations with few data points to complex/rich visualizations with increased data points) and time (engagement duration) across an explanatory-exploratory visualization task Venn diagram.

The proposed HI-viz is a unique visualization type in time-critical contexts (Figure 1). Characteristics include heightened complexity with the presentation of multiple discrete data points, similar to a dashboard, but in a single integrated visual representation. It requires rapid processing, similar to glanceable visualizations, but with this being the primary task and requiring additional engagement. It can link and communicate relationships between information points, similar to diagrammatic representations, but in a curated, coherent, and concise manner. The requirements of this visualization type transition beyond the pre-attentive—a rapid subconscious processing of information in the visual environment where the viewer is drawn to elements of the design or data through pre-attentive attributes (color, form, movement, and spatial positioning) [10]—and “glanceable” timescales into a nuanced attentive area we define as “rapid attention processing.” This stipulated segment of the timescale typically features glanceable visualizations and individual chart types that are usually lower in information density and complexity. Thus, the holistic integrated visualization has the potential to convey more information in a shorter time frame through a single visual representation (Figure 2).

The synthesis of the theoretical underpinning (Figure 2) positions the information visualization types against *information* (from simple visualizations with few data dimensions to complex and rich visualizations with increased data dimensions) [18,23,42-44,47,48,50-52] and *time* (engagement duration) [18,21,23-26,33,47,48,50-55,62], across an explanatory-exploratory visualization task Venn diagram [43,49]. While reading the chart (Figure 2), one should keep in mind that it communicates the relative time and information complexity of each visualization type theoretically and provides a general comparison as synthesized through analysis of the literature. The authors have positioned visualization types considering information complexity (less or more information—data dimensions) and time (to understand the information). In practice, how the visualization types perform may vary depending on their physical implementation and information complexity.

**Figure 3:**
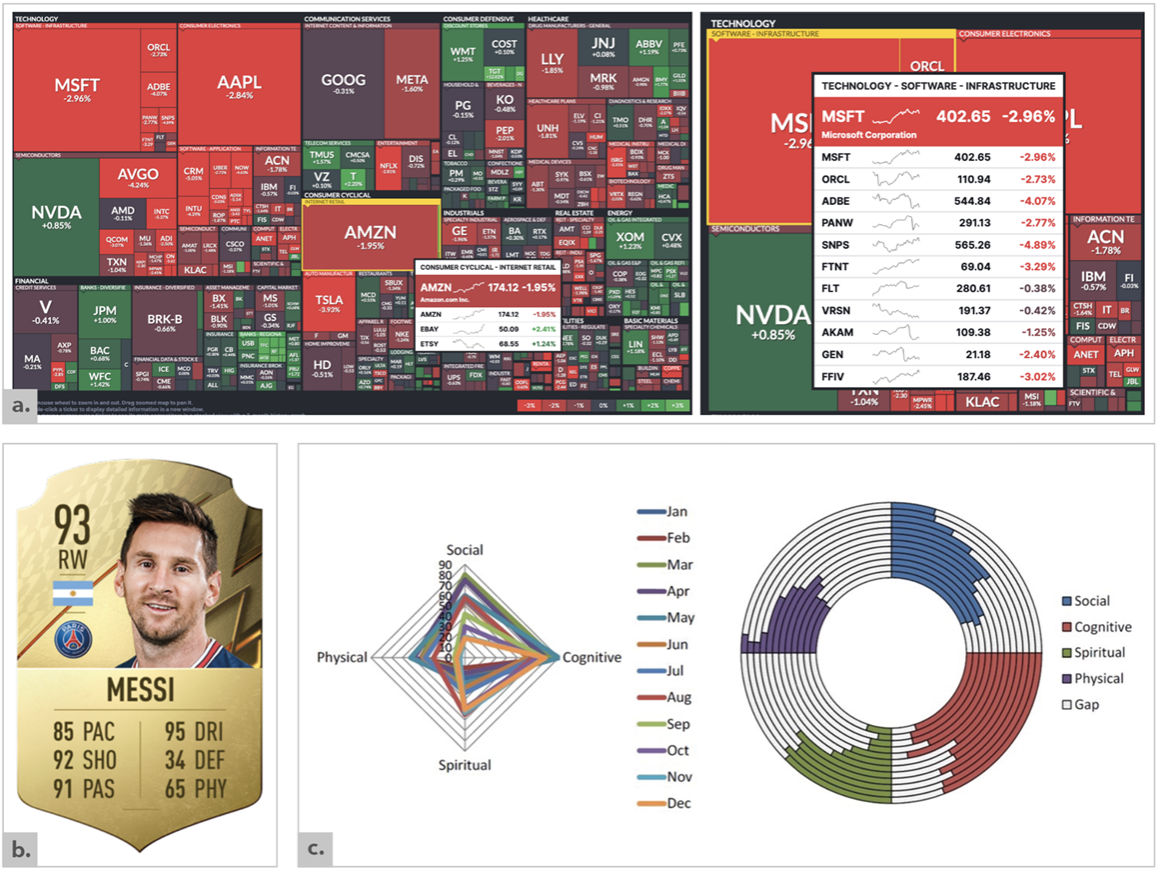
(A) Finviz [64]; (B) FIFA 22 player rankings [63]; (C) Older Adults' Wellness by Le et al [4].

Examples of HI-viz in other domains such as sports, finance, and military applications have proven effective in giving a quick at-a-glance presentation of multivariate data providing both summary and context. Profiles of football players in fantasy leagues and computer games provide a holistic overview of key information regarding the professional athlete and their skills and abilities (eg, FIFA 22 video game player rankings [63]). The holistic integrated profile of Messi (Figure 3B [63]) quickly informs the viewer that the player is an excellent (93 overall aggregated score) male football player of Argentinean nationality; plays for Paris Saint-Germain Football Club (Paris) at club level; and excels at physical, mental, and technical attributes such as shooting, passing, and dribbling. This information captures a holistic overview of the player and their attributes. This FIFA 22 player card presents 12 core data dimensions pertaining to the player (name, photo, overall rating, position, nationality, club, and 6 individual performance attribute statistics) in a single integrated “player profile” visualization. The categories and numerical values are easily interpretable, allowing the viewer to understand player strengths and weaknesses as well as facilitating easy comparison between players holistically and across categories.

Similarly, Finviz [64], an interactive tree map of the stock market, provides users with an at-a-glance holistic summary of stocks categorized by industry and sectors against key performance metrics (price fluctuation and market cap). This single integrated view allows the viewer to see the overall market, how stocks compare within a sector, and relative sector performance. Color coding (red for losses and green for gains) and the size of the stock tiles immediately illustrate stock performance, performance across the market, and whether a sector is up or down. This visualization is high in information complexity, with hundreds of stocks and each tile communicating the stock ticker symbol, price change (percentage up or down), and market dominance (the size of the tile) within its respective sector and industry groupings.

Military aircraft, too, amalgamate high information complexity and display a user interface for rapid information processing. Flight panels integrate disparate data dimensions such as altitude (above ground level), flight path, aircraft pitch and roll, current airspeed, vertical speed, aircraft heading, engine status (speed, fuel, temperature, and torque), outside air temperature, navigation data, alerts, and flight warnings. This provides an at-a-glance holistic overview for the pilot in a single display for rapid comprehension and decision-making. The integrated view enables the layering and combining of different types of information, allowing the pilot to track and process core information simultaneously.

Within health care, Le et al [4,65] present “an approach to capture older adults’ wellness” through 3 alternate visualization techniques (stacked bar charts, polygons, and donut displays) in addition to an integrated measure of wellness—cognitive, physiological, social, and spiritual. This presents alternate approaches, nonexistent in electronic systems, combining the 4 factors into 1 graphical representation of overall wellness (Figure 3C [4]). The tool captures numerous data points pertaining to older adults’ wellness over a 12-month period, with the holistic overview enabling quick interpretation of wellness indicators (physiological, social, spiritual, and cognitive) and correlations at a glance in a single integrated visual representation. The aim to provide additional granularity for health information, while maintaining the representation of overall wellness was an interesting paradigm, offering the viewer a quick overview in addition to further detail and layers of information. Similarly, a holistic integrated visualization in primary care can inform us, for example, that we are dealing with a complex older adult female patient who is obese, smokes, and has uncontrolled diabetes and hypertension. The information could be derived from different sources of data (ie, electronic health records, wellness sensors, and secondary care) and integrated into a single visual representation, and relationships between data dimensions could be highlighted through links (ie, medications and health problems or medications and allergies).

### Motivation: Designing HI-viz

Guidelines and principles are useful for the systematic and effective creation of novel outputs following established tasks, techniques, and taxonomies [13,66]. Numerous studies have reviewed or presented guidelines for visualizations more broadly [5,7-10]. Their findings include overall design processes, principles, or frameworks for information visualization [9,11-13,66-69]; visualization taxonomies [70-78]; the grammar of graphics [10,79-81]; designing for storytelling [58,82-89]; designing dashboards [14-16]; multiple views [90,91]; designing infographics [58,84,86,92-95]; and glanceable interfaces [17,18]. Kohlhammer and Zeltzer [20] proposed a visualization architecture for time-critical contexts, aiming to merge support for decision-making with situational awareness. Their paper touches on the design of a user interface module and a decision-centered visualization architecture, which shows the connections between modules (data access, decision focus, user interface, and submodules). However, no approaches, methods, principles, or guidelines were provided toward designing holistic integrated visualizations for time-critical contexts.

To design HI-viz, as the needs and constraints differ from those of other visualization use cases (Figures 1 and 2), a contextually appropriate set of guidelines is required combining established guidelines and principles for rapid processing, integrated presentation, and a holistic understanding.

## Methods

We systematically searched the literature for peer-reviewed research on visualization strategies, guidelines, and taxonomies. The literature selection followed the PRISMA (Preferred Reporting Items for Systematic Reviews and Meta-Analyses) guidelines, an established method for systematic review of scientific publications (Multimedia Appendix 1) [96,97].

The following 6 databases were searched for published articles in peer-reviewed journals: ACM Digital Library, Google Scholar, IEEE Xplore, PubMed, Scopus, and Web of Science. The search was up to August 2024 using the terms (“visualisations” OR “visualizations”) AND (“guidelines” OR “taxonomy” OR “taxonomies”), with studies restricted to the English language. The search terms were intentionally broader to ensure that all relevant publications pertaining to visualizations, guidelines, and taxonomies were captured. The Boolean logic ensured that the search results included publications pertaining to visualizations (with both the UK and US spellings) as well as a minimum of 1 related concept (guidelines, taxonomy, or taxonomies). Search terms were screened across publication titles and abstracts.

Papers were included if they were published in English and related to strategies and methods (guidelines and taxonomies) for designing visualizations and if the guidelines related to one or more of the following: *holistic understanding for complexity* (designing visualizations for multidimensional data), *time* (ie, time-critical applications and designing for quick information processing), and *integrating* multidimensional data within a single visual representation. References from the included articles were examined to identify additional peer-reviewed and industry literature.

Papers were excluded if (1) they were duplicates; (2) they were not in English; (3) they focused on design guidelines for specific applications, such as augmented reality, virtual reality, or mixed reality, where the guidelines or taxonomies did not address integrated presentation; (4) they did not involve integrating or combining multidimensional data into a single visual representation; (5) they did not focus on guidelines for visualizing complex multidimensional data for holistic understanding; or (6) they did not address designing for rapid information processing.

**Figure 4:**
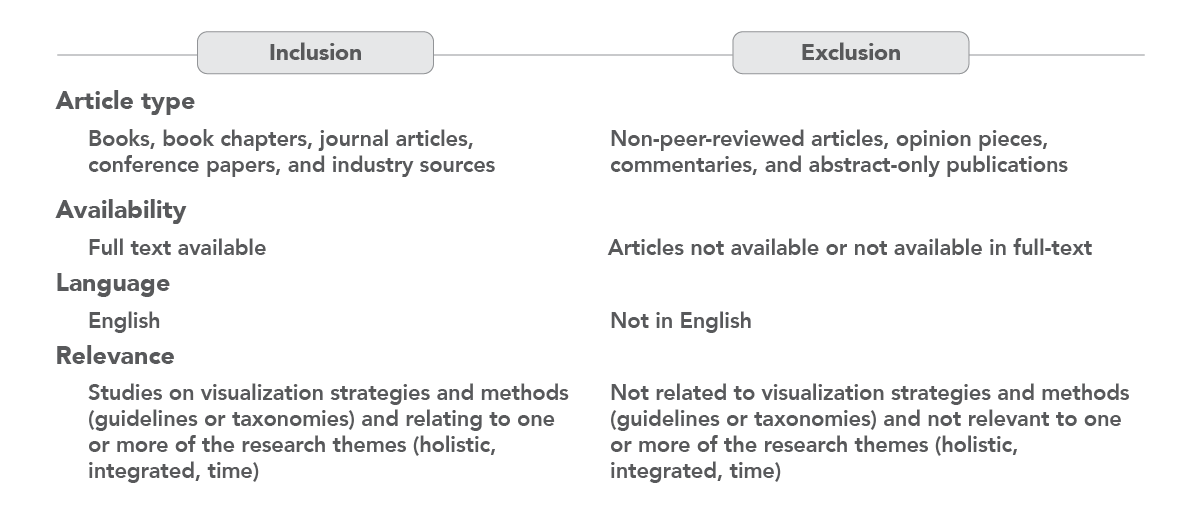
Study Inclusion and Exclusion Criteria.

Following title and abstract screening, the full texts of the remaining papers were checked for eligibility against the inclusion and exclusion criteria (Figure 4). The first author undertook the study search and screening for exclusion and inclusion of studies, which was confirmed to be accurate and complete by another reviewer (TP). Throughout the screening process, coauthors were consulted as needed in determining the eligibility of the studies.

Through an inductive thematic analysis approach, themes emerging from the data were identified. Initial codes were elected by the first author. NVivo (version 12; QSR International) was used to code and analyze the data. Codes were discussed, grouped into broader themes, and iteratively reviewed among the 3 authors to reach a consensus.

## Results

### Overview

**Figure 5:**
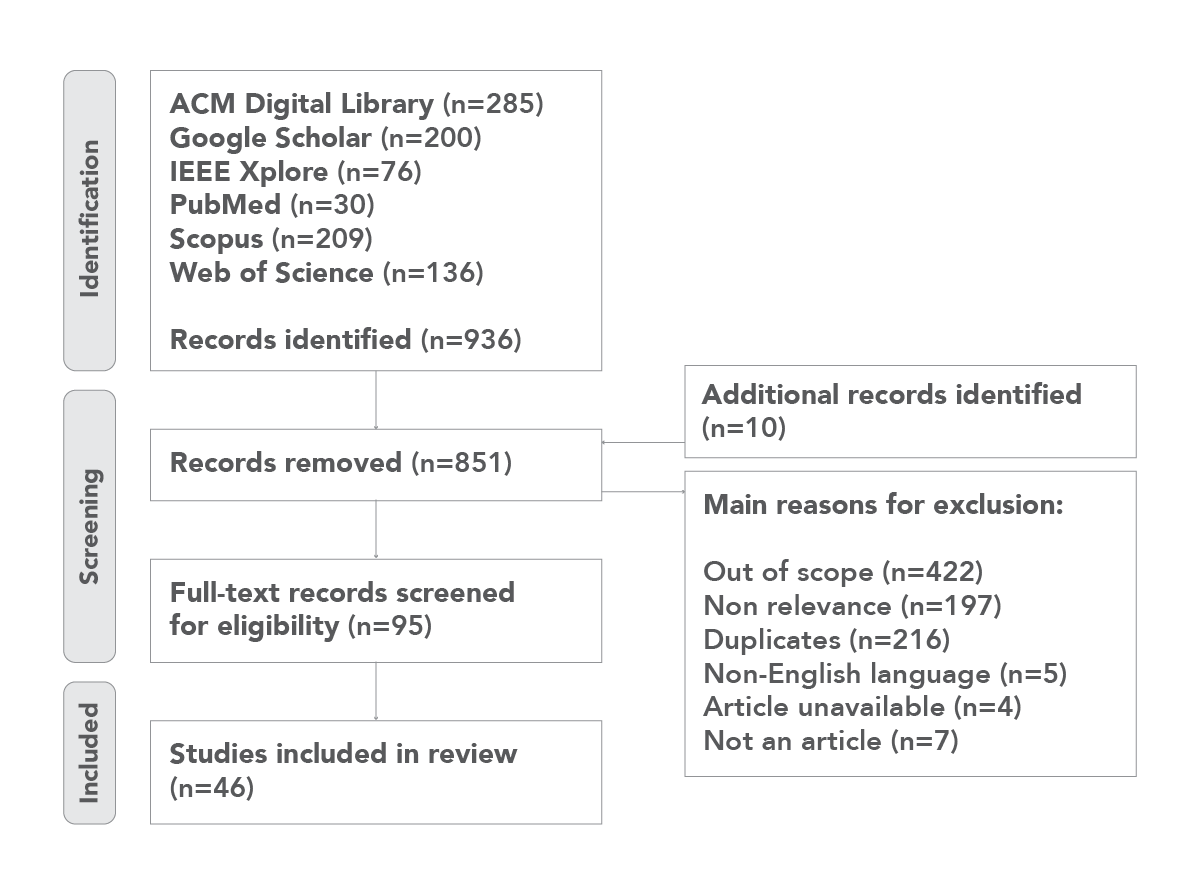
PRISMA (Preferred Reporting Items for Systematic Reviews and Meta-Analyses) flow diagram—flow of information through the different phases of the systematic review.

The systematic literature search resulted in a pool of 936 publications pertaining to visualization guidelines and taxonomies. A total of 90.9% (851/936) of the papers were excluded based on duplicity, non-English language, and title and abstract screening. In total, 58% (49/85) of the remaining articles were excluded after reading the full text. A total of 36 papers met the inclusion criteria [9,17,67,69,71,90,98-127]. A further 10 publications were included upon examining references from included articles [10,19,128-133], including 2 from industry sources [5,134]. A total of 46 papers were included in the final review (Figure 5).

In total, 48% (22/46) of the papers were related to visualizing complexity to provide an overview or *holistic* understanding of multidimensional data (ie, prioritization of information, information reduction, organization of information, abstraction representations to communicate information, visual aggregates, and interacting with visualizations or to call and filter information); 28% (13/46) of the papers focused on *integrated presentation*, integrating or combining multidimensional data into a single visual representation (ie, through aggregation of data, integration of numerical and statistical values, and grouping approaches [ie, gestalt]); and 35% (16/46) of the papers pertained to *time* and designing for rapid information processing (ie, implementing pre-attentive processing attributes and features, gestalt principles, glanceable display guidelines, information abstraction, and interaction; minimizing task complexity; and enhancing situational awareness). A total of 65% (30/46) of the papers presented *general* information visualizations or visual communication guidelines and principles. In this category, we included more universal user-interface and user experience guidelines as well as considerations of information reduction, clarity of communication, information organization, interaction and navigation, relationships among and between information, minimization of task complexity, and alarm support and projection. Some of the papers (31/46, 67%) addressed more than one attribute (Table 1). A detailed mapping of the features of the holistic, integrated, time, and general guideline characteristics of the studies in this review is mapped in Multimedia Appendix 2 [5,10,17,19,67,69,71,90,98-134].

**Table 1:** Characteristics of the studies: holistic, integrated, time, and general design.

Study, year	Holistic (n=22)	Integrated (n=13)	Time (n=16)	General design (n=30)
Adnan et al [98], 2008	✓			✓
Ajani et al [99], 2022				✓
Andrews et al [100], 2011	✓	✓		✓
Bateman et al [101], 2010			✓	✓
Brath [102], 1997	✓			✓
Chang and Nesbitt [103], 2006		✓		✓
Cowan [104], 2015	✓			✓
De Carlo et al [105], 2022	✓			✓
De Weck et al [106], 2011				✓
Dix [107], 2013	✓			✓
Dybala et al [108], 2020		✓	✓	
Ellis and Dix [109], 2007				✓
Elmqvist and Fekete [9], 2010	✓	✓		
Endsley [19], 2012	✓			✓
Fekete and Plaisant [110], 2002	✓		✓	
Forsell and Johansson [69], 2010			✓	✓
Healey et al [111], 1995			✓	
Hick [128], 1952			✓	✓
Idrissov et al [112], 2020		✓		✓
Kelleher and Wagener [113], 2011		✓		✓
Kornhauser et al [114], 2009		✓	✓	✓
Krekhov et al [115], 2019			✓	
Ku et al [116], 2012	✓			
Lima [5], 2019			✓	✓
Matthews et al [17], 2006	✓		✓	✓
Midway [67], 2020	✓			✓
Miller [129], 1956	✓			✓
Moran [134], 2016	✓			✓
Moreira et al [117], 2020		✓	✓	
Murray et al [118], 2017				✓
Perer and Shneiderman [119], 2009	✓	✓		
Rechtin [120], 1991	✓			✓
Rodrigues et al [121], 2006	✓		✓	
Rosli et al [122], 2015		✓		
Seong and Nuamah [123], 2020		✓	✓	
Shaalan and Jusoh [124], 2020				✓
Shneiderman [71], 1996	✓			✓
Spence [130], 2001	✓		✓	✓
Triesman [131], 1985			✓	
Tufte [132], 1983				✓
van Ham and Perer [125], 2009	✓			
Wagemans et al [126], 2012		✓		
Wang Baldonado et al [90], 2000	✓			
Ware [10], 2020		✓	✓	
Wenzel et al [127], 2003				✓
Yablonski [133], 2020	✓			✓

This systematic review highlights that, presently, no specific guidelines or principles were found to address all the characteristics of HI-viz, namely, holistic, integrated visualizations for time-critical contexts. Consideration of the established principles, guidelines, and taxonomies from the fields of visualization, design, and cognitive science and a combination thereof may aid in the design of holistic integrated visualizations for time-critical contexts. A summary of the key guidelines and principles identified in this review was extracted, collated, and categorized in Table 2. The categories in Table 2 were created through the following steps:

1. Repeated features were combined across the 4 characteristics identified in this systematic review (ie, gestalt principles, reduction, organization, abstraction, aggregates, and task complexity).
2. Interaction was deconstructed to provide prescriptive categories (ie, experience, overview, details on demand, and rearrangement).
3. Some guidelines were broad and closely aligned with existing categories. For example, the 4 guidelines pertaining to “glanceable” displays better aligned with organization, abstraction, visual clarity, and experience. Where the user experience or system principles (Hick’s law, Miller’s law, and Tesler’s law) aligned with more than one feature identified in this systematic review, they were better mapped across organization, reduction, and task complexity.
4. Individual unique features identified in this systematic review were maintained (ie, pre-attentive, prioritization, relationships, navigation, alarms, and visual clarity).

**Table 2:** Summary of key established design and visualization guidelines and principles for holistic integrated information visualizations (HI-viz).

Category, guideline, and principle			References
**Pre-attentive**			
	G01. Use pre-attentive attributes (color, form, spatial positioning, and movement)	Ware [10], Fekete and Plaisant [110], Healey et al [111], Kornhauser et al [114], Krekhov and Kruger [115], Moreira et al [117], Rodrigues et al [121], Seong et al [123], Spence [130], Triesman [131]	
**Prioritization**			
	G02. Prioritize the core information and message to be communicated	Midway [67]	
	G03. Manage featurism through prioritization and flexibility	Endsley [19]	
	G04. Say no to feature creep—buck the load	Endsley [19]	
**Reduction**			
	G05. Remove the extraneous	Dix [107], Forsell and Johansson [69]	
	G06. Consider dataset reduction—efficiency and ease of use of the dataset	Forsell and Johansson [69]	
	G07. Use information filtering carefully	Endsley [19]	
	G08. Explicitly identify missing information	Endsley [19]	
	G09. Reduce display density but do not sacrifice coherence	Endsley [19]	
**Organization**			
	G10. Organize and group information around goals	Endsley [19], Cowan [104], Miller [129], Moran [134]	
	G11. Ensure spatial organization for user navigation—the distribution of elements	Forsell and Johansson [69]	
	G12. Ensure that consistency in design choices is maintained in similar or different contexts	Forsell and Johansson [69], Chang and Nesbitt [103]	
	G13. Maintain consistency in design elements that are similar	Lima [5], Matthews et al [17]	
	G14. Provide consistency and standardization on controls across different displays or systems	Endsley [19]	
	G15. Ensure logical consistency across modes and features	Endsley [19]	
	G16. Consider Miller’s law of “seven, plus or minus two” for grouping information	Miller [129], Moran [134], Cowan [104]	
	G17. Remove clutter and “chart junk”	Ajani et al [99], Bateman et al [101], Ellis and Dix [109], Tufte [132]	
	G18. “Relationships among elements are what gives systems their added value”	Rechtin [120], Andrews et al [100], Dix [107], Idrissov et al [112], Murray et al [117]	
**Gestalt principles**			
	G19. Proximity—elements that are close together are perceptually grouped together	Ware [10], Chang and Nesbitt [103], Idrissov et al [112], Kornhauser et al [114], Moreira et al [117], Rosli and Cabrera [122], Seong et al [123], Wagemans et al [126]	
	G20. Similarity—similar elements tend to be grouped together	Ware [10], Chang and Nesbitt [103], Dybala et al [108], Idrissov et al [112], Kornhauser et al [114], Moreira et al [117], Rosli and Cabrera [122], Seong et al [123], Wagemans et al [126]	
	G21. Connectedness—linked elements express a relationship between them	Ware [10], Idrissov et al [112], Seong et al [123], Wagemans et al [126]	
	G22. Continuity—connections between visual elements are discernible if they are smooth and continuous	Ware [10], Chang and Nesbitt [103], Dybala et al [108], Idrissov et al [112], Moreira et al [117], Seong et al [123], Wagemans et al [126]	
	G23. Symmetry—symmetrical elements are perceived as members of 1 group	Ware [10], Idrissov et al [112], Seong et al [123], Wagemans et al [126]	
	G24. Closure—elements with a closed contour are perceived as shapes or object-like and contours with gaps are perceptually closed.	Ware [10], Chang and Nesbitt [103], Dybala et al [108], Idrissov et al [112], Moreira et al [117], Rosli and Cabrera [122], Seong et al [123], Wagemans et al [126]	
	G25. Common region—elements within a contour or boundary are perceived as a group.	Ware [10], Idrissov et al [112], Moreira et al [117], Seong et al [123], Wagemans et al [126]	
	G26. Relative size—smaller elements inside larger elements are perceived as separate objects	Ware [10], Idrissov et al [112]	
	G27. Common fate—(in motion perception) elements moving together in the same direction tend to be perceived as a unified group	Ware [10], Chang and Nesbitt [103], Dybala et al [108], Idrissov et al [112], Kornhauser et al [114], Moreira et al [117], Seong et al [123], Wagemans et al [126]	
**Abstraction**			
	G28. Communicate the fundamental features of what is being designed clearly and accurately	Endsley [19], Forsell and Johansson [69], Cowan [104], De Weck et al [106], Dix [107], Miller [129], Moran [134]	
	G29. “Use abstraction representations” to distil and emphasize key aspects of the information in its most essential detail via iconic representations to communicate the information	Matthews et al [17], Bateman et al [101]	
	G30. Consider Tesler’s law: for any system, there is a certain amount of complexity that cannot be reduced	Yablonski [133]	
**Aggregates**			
	G31. Entity budget—maintain an entity budget by controlling the quantity of data rendered on-screen to avoid visually overloading the viewer	Elmqvist and Fekete [9]	
	G32. Visual summary—aggregates should convey information about underlying data	Elmqvist and Fekete [9], Andrews et al [100]	
	G33. Visual simplicity—aggregates should be clean and simple	Elmqvist and Fekete [9], Kelleher and Wagener [113]	
	G34. Discriminability—aggregates should be distinguishable from data items	Elmqvist and Fekete [9]	
	G35. Fidelity—beware that abstractions may lie	Elmqvist and Fekete [9]	
	G36. Interpretability—aggregate items only so much so that the aggregation is still correctly interpretable within the visual mapping	Elmqvist and Fekete [9]	
**Visual clarity**			
	G37. Ensure that visuals are simple, well-understood representations that can be grasped when looking for the first time	Dix [107], Midway [67], Kelleher and Wagener [113], Kornhauser et al [114], Tufte [132]	
	G38. “Make visuals distinct,” which helps improve the identification of elements and draws the viewer’s eye to new or updated information	Matthews et al [17]	
**Alarms**			
	G39. Do not make people rely on alarms; provide projection support	Endsley [19]	
	G40. Support alarm confirmation activities	Endsley [19]	
	G41. Make alarms unambiguous	Endsley [19], Andrews et al [100]	
	G42. Ensure that alarms do not disrupt ongoing activities	Endsley [19]	
	G43. Support the assessment and diagnosis of multiple alarms	Endsley [19]	
**Experience**			
	G44. “Match user expectations,” which will reduce learning effort	Matthews et al [17]	
	G45. Experience with the visualization should be intuitive and easy	Lima [5]	
	G46. Interactivity should be driven by user needs	Spence [130], Lima [5]	
**Overview**			
	G47. Gain an overview of information	Shneiderman [71], Andrews et al [100], De Carlo et al [105], Dix [107], Elmqvist and Fekete [9], Fekete and Plaisant [110], Ku et al [116], Van Ham and Perer [125]	
	G48. Zoom in on items of interest	Shneiderman [71], Wang Baldonado et al [90], Andrews et al [100], De Carlo et al [105], Dix [107], Fekete and Plaisant [110], Perer and Shneiderman [119]	
	G49. Filter out uninteresting items	Shneiderman [71], Wang Baldonado et al [90], Adnan et al [98], Fekete and Plaisant [110], Perer and Shneiderman [119], Van Ham and Perer [125]	
**Details on demand**			
	G50. Details on demand; select an item or group and obtain information when needed	Shneiderman [71], Wang Baldonado et al [90], Adnan et al [98], Andrews et al [100], Brath [102], De Carlo et al [105], Fekete and Plaisant [110], Van Ham and Perer [125]	
	G51. Embrace dynamic, fast, and clever experiences; a hover can reveal information faster than clicking to open and clicking to close	Lima [5], Ware [10]	
**Relationships**			
	G52. View relationships between items	Shneiderman [71], Wang Baldonado et al [90], Andrews et al [100], Kelleher and Wagener [113], Ku et al [116], Perer and Shneiderman [119]	
	G53. Keep a history of actions to support undo, replay, and progressive refinement	Shneiderman [71]	
	G54. Strong contextual cues aid in navigating through the visualization and going back	Lima [5], Andrews et al [100]	
	G55. Allow for the extraction of subcollections and of the query parameters	Shneiderman [71]	
**Task complexity**			
	G56. Minimize task complexity and ensure minimal actions with respect to the number of actions necessary to accomplish a goal or task	Endsley [19], Forsell and Johansson [69]	
	G57. Flexibility—refers to the number of possible ways of achieving a goal	Forsell and Johansson [69], Chang and Nesbitt [103]	
	G58. Prompting—refers to all means that help know all alternatives when several actions are possible	Forsell and Johansson [69]	
	G59. Consider Hick’s law, which states that “the time it takes to make a decision increases with the number and complexity of choices”	Hick [128]	
**Rearrangement**			
	G60. View the visualization from multiple angles or perspectives	Spence [130], Brath [102], De Carlo et al [105], Dix [107], Fekete and Plaisant [110], Perer and Shneiderman [119], Rodrigues et al [121]	

### Proposed HI-viz Guidelines

#### Overview

The 60 guidelines and principles extracted from this systematic review in Table 2 were grouped into 5 main interlinked guidelines for designing HI-viz. Through an inductive thematic analysis approach, 16 top-level categories emerging from the data were identified (Multimedia Appendix 2 and Table 2). Related guidelines were thematically grouped through the top-level categories and underlying principles that linked them, capturing the essence of each group of guidelines (ie, guidelines pertaining to *prioritization* and *reduction* of information were grouped with the common focus of prioritizing key information and reducing the extraneous). The categorization was iteratively reviewed and corroborated among all authors to reach a consensus.

The detailed descriptive and prescriptive guidance references the 60 theoretical guidelines (G01-G60) below.

#### Prioritize Core Information to Obtain a Holistic Overview of Task-Related Information

To ensure that a visualization provides a holistic overview for a specific task, prioritize the core information, establish hierarchy, reduce clutter, filter information carefully, and use visual cues to highlight important elements and improve user efficiency. Specifically, to attain a holistic overview, a hierarchy must be established in the dataset and design. The core information, message to be communicated, and features must be prioritized (G02 and G03), and the importance between them must be affirmed to aid the management of information in the visualization as well as reducing cognitive complexity on the user (G03). To convey hierarchy and rapidly call attention to prioritized information elements or areas, pre-attentive attributes such as color, form, spatial positioning, and movement should be used (G01). Extraneous information and features that are not core to the task and infrequently accessed must be identified and reduced (G05). This will prevent “feature creep,” which is the gradual addition of nonessential features that can complicate the user experience (G04), and limit distractions from the core information needs and wants, contributing to the efficiency and ease of use (G06). The filtering of information must be used carefully (G07), reducing display density to support information “at a glance” but not sacrificing coherence (G08 and G09) or augmenting the need to navigate and find information, consequently adversely increasing the time taken for information assimilation (G07). Thus, missing information must also be explicitly identified to highlight what is unavailable or not unimportant (G08) and preventing the user from having to search and navigate for data that are not provided.

#### Organize the Constituent Visualization Elements to Provide an Integrated Representation

To ensure an integrated visualization, organize information around the user’s core goals and structure information effectively using grouping, layering, and visual principles into a single connected and combined representation. Specifically, to combine and integrate the constituent elements into a single integrated representation, focus and organize information around the users’ core goals and provide the required information directly (G10). Ensure an efficient spatial distribution of information and features to aid user navigation (G11). Organize information by importance and commence with a holistic overview (macro) that captures and communicates the core message, subsequently implementing progressive disclosure to provide detail (micro) and context on demand.

Integrate the constituent elements into the data and design through “chunking” or grouping information while considering Miller’s law of “seven, plus or minus two” as a limit for grouped information (G16). Use gestalt principles of organization (proximity, similarity, connectedness, continuity, closure and common region, and symmetry) to guide the arrangement and structuring of information into a connected, combined, and integrated whole (G19-G27). In layering information, use “visual aggregation” (sum or count, average, mode, extents, medians, percentiles, and distribution) to communicate information about underlying contents while not misrepresenting the data in the process of simplification (G31-G36), as well as abstraction to distil and emphasize key aspects of the information in its most essential detail via iconic representations to aid in the quick identification of information (G29). Consider Tesler’s law: for any system, there is a certain amount of complexity that cannot be reduced (G30). Establish and communicate relationships between information and integrated elements through links and connections to guide insights and provide “added value” (G18).

#### Maintain Consistency and Clarity Across All Elements and Interactions to Ease Cognitive Demand

To reduce cognitive load when using the visualization, maintain consistency and clarity across design elements and interactions by standardizing attributes, modes, and features; simplifying visual elements; and using visual cues for effective notifications. Specifically, maintain consistency across similar design elements (G13), visual and spatial attributes (color and geometry [shape, size, scale, and position]; G11 and G12), and modes and features (G14, G15, and G28) to build familiarity and help manage complexity. Provide standardization across layout, format, and labels to reduce the time taken to search as well as helping develop and build a mental model to assimilate new or different information across the visualization (G14). Ensure clarity in the visual representation by eliminating extraneous elements and embellishments (G17); thus, every entity supports data insights. Develop simplicity in visual elements with well-understood representations that can be grasped when looking for the first time (G37) while being distinct to help aid the identification of elements and drawing the viewer’s eye to new or updated information (G38). To rapidly call attention to new or updated information, use pre-attentive attributes (color, form, spatial positioning, and movement; G01). Communicate and provide updated or missing information and factors through discernible “alarms” or notifications (G40, G41, and G43), ensuring that these do not disrupt ongoing activities (G42) but provide projection support (G39).

#### Use Familiar Mental Models to Enable Intuitive Interactions

To facilitate intuitive interactions, design interfaces based on familiar mental models, use established affordances and contextual clues, and simplify tasks to reduce cognitive effort. Specifically, design the interface with existing mental models driven by user needs (G46). Use familiar, established interaction affordances to support intuitive use (G45) and reduce learning effort (G44). Minimize task complexity by ensuring a minimal number of actions to accomplish a goal or task (G56 and G59) and provide flexibility to communicate the number of possible ways to achieve a goal (G57), prompting with strong contextual clues to help know the alternatives when several actions are possible (G58). Support navigation by allowing users to undo actions, replay previous steps, and progress through the visualization (G53 and G54) and consider Hick’s law, which states that “the time it takes to make a decision increases with the number and complexity of choices” (G59).

#### Afford Depth of Data Retrieval by Enabling Users to Zoom Into the Data

To ensure efficient data retrieval, design the visualization to allow users to navigate between overview and detailed information on demand, communicate relationships, and view data from multiple perspectives. Specifically, use established interactive approaches to minimize complexity. Commence with a holistic overview of the data (G47) and provide progressive disclosure through interactions, including zooming in on items of interest (G48), details on demand to obtain information when needed (G50), and a filter for uninteresting items (G49). A hover can reveal information faster than clicking to open and clicking to close (G51). Enable the user to rearrange or view the visualization from different perspectives (G60) and link elements to communicate the relationship between items (G52), guiding analytical reasoning and insights. Support the extraction and retention of information desired by the user once they have attained it (G55).

## Discussion

### Principal Findings

In this paper, we argue and establish that, in time-critical contexts, holistic visualizations that integrate multidata variables into a single visual representation are a distinct information visualization type with different needs and constraints. We position this visualization, called a HI-viz, in a 2D plot (time and information; Figure 2), highlighting that HI-viz has the potential to convey more information in a shorter time frame. HI-viz in time-critical contexts requires approximately 30 seconds for information assimilation and processing, applying both pre-attentive and attentive processing (ie, requiring more engagement than glanceable displays but being quicker than dashboards) and integrating relatively large amounts of data (ie, similar in complexity to dashboards but as an integrated whole). This systematic review across 6 databases (ACM Digital Library, IEEE Xplore, PubMed, Scopus, Web of Science, and Google Scholar) confirms that, currently, no specific guidelines or principles were found to address all the characteristics of HI-viz, namely, holistic, integrated visualizations for time-critical contexts. As the needs and constraints differ from those of other visualization types, a contextually appropriate set of guidelines is required, combining established principles, guidelines, and taxonomies from the fields of visualization, design, and cognitive science to aid in the design of HI-viz for time-critical contexts.

### HI-viz Guidelines

The 5 proposed HI-viz guidelines, derived from existing design theories and guidelines, are meant to provide design directions for practitioners and researchers working on HI-viz use cases based on theoretical grounding. Our work intentionally sought to offer practical prescriptive support. For instance, we focused on design guidelines that have observable properties (such as “make icons visually distinct”) and excluded broad or conceptual principles (such as “less is more”). Such practicality is also shown through the adaptation from other known lists of guidelines (eg, the “Six Principles for Designing any Chart” by Lima [5] and “Four Principles for Glanceable Displays” by Matthews et al [17] that focus on conceptual and specific principles for visualizations and displays) together with contextual suggestions, resulting in grounded direction.

The first HI-viz guideline emphasizes the need to prioritize core information and reduce extraneous information for a holistic overview of the data. Excessive details and features irrelevant to the task can contribute to the cognitive overload and overwhelm the user [69,107], consequently reducing situational awareness and negatively impacting decision-making. Thus, reducing “visual clutter” [19] and establishing hierarchy in the data (ie, what is most important) [19,67] and design allows the user to quickly obtain the “bigger picture” and focus on the key information for rapid comprehension without sacrificing the depth of understanding. In time-critical contexts in which users rely on complex data, this facilitates a timely review of what is most important without having to switch between multiple displays or areas to assimilate information.

The second HI-viz guideline pertains to the integrated representation of information through structuring and organizing the diverse data dimensions. With larger datasets and higher information complexity, the grouping of information [104,129,134] and use of gestalt principles of organization [10,103,108,112,114,117,122,123,126] can bring together disparate data, facilitating patterns in groups over parts. Links and connections between information and elements provide the “added value” [100,107,112,118,120], facilitating relationships between data points in a concise and curated manner and, thus, distinguishing HI-viz from dashboards through the connected and integrated whole. Revisiting Finviz (Figure 3A [64]), the respective sector and industry groupings of stocks immediately illustrate stock performance across the market and whether a sector is up or down. This also resonates and aligns with Miller’s law of “seven, plus or minus two” for grouping information [104], reducing cognitive overload and enabling the recall of information considering the capacity of working memory.

The third HI-viz guideline emphasizes consistency across all elements (design and interaction) and effective notifications to call attention to new or updated information. Implications of consistency across visual and spatial attributes allow the user to build familiarity and comprehend the information [19,69,103,104,106,107,129,134]. In time-critical domains such as clinical settings, inconsistent information communication can lead to misdiagnosis, incorrect treatments, and patient harm. Effective notifications can allow the user to quickly understand what information is new, updated, or missing [19,100] (eg, whether a patient’s blood pressure results are recent or normal, high, or low; whether the data is old or missing data; and whether an up-to-date reading is required), improving task efficiency, accuracy, and user performance.

The fourth HI-viz guideline emphasizes the need for using familiar mental models for intuitive interactions. Implementing known affordances and contextual clues [5] ensures that the user requires minimal training, onboarding, and support. In a time-critical context, this contributes toward the efficient navigation and completion of the task [19,69,128]. The user knows how to complete the task; the number of possible ways and alternatives [69]; and how to undo, replay, and progress [5,71,100]. Thus, as per Hick’s law [128], task complexity is reduced by limiting the choices and steps required, which can improve situational awareness, decision speed, and accuracy.

Here, a question arises on how user knowledge and familiarity with the data, visualization type, and task at hand influences information processing speed. For instance, the rapid comprehension of HI-viz might be strongly linked to a user’s previous experience with the data and the specific visualization format used. Numerous research has shown that training or experience with the data or a specific format can impact the user’s ability to understand and interact with the visualization and make decisions, consequently enhancing accuracy and efficiency [135-140]. Cognitive factors such as spatial and perceptual abilities, which differ between users, also contribute to task performance [141-145]. Future work can explore how different user groups (experts vs novices) interact with HI-viz and how previous knowledge, experience, or familiarity can impact task efficiency or information comprehension. What formats or modes of interaction cater best to varying levels of expertise? What level of detail is required for users to gather the core information?

The fifth HI-viz guideline references efficient data retrieval by allowing users to zoom in and interact with information on demand. This extends the information-seeking mantra by Shneiderman [71]—“overview first, zoom and filter, then details on demand”—a seminal principle in the design of interactive visualizations. For HI-viz, this approach affirms the explanatory-exploratory positioning whereby users can navigate between the at-a-glance holistic overview and the detailed contextual information to attain insights across multiple layers of information [9,71,90,100,105,107,110,116,119,125]. Rearranging views or viewing data from multiple perspectives [102,105,107,110,119,121,130] can help communicate relationships within and between data dimensions and points [71,90,100,113,116,119]. Smart interactions, such as a hover [5,10], reduce unnecessary clicking between areas of the interface to gather and make sense of information (ie, reducing “click-fatigue” [1]) and, thus, facilitating quicker access to core information for task completion.

These guidelines provide a foundation for designing HI-viz that can contribute to improved situational awareness and informed decision-making [3] as well as enhancing user experience and improving information comprehension.

However, there is a trade-off between generality and specialization of guidelines [146], and therefore, these guidelines might not adequately support all time-critical domains. For example, additional specialized guidelines may be required in certain scenarios, such as in aviation, cybersecurity, finance, and military contexts. Thus, while we believe that we captured a broad range of key and relevant guidelines from both industry and academic literature as we searched the literature in a systematic way, this study acknowledges that the guidelines are derived from these databases and it is possible that other relevant guidelines to the design of HI-viz were missed. Such guidelines can be improved as future work continues to explore additional applications HI-viz, which may also highlight other theories. These guidelines serve as a starting point for designing holistic integrated visualizations in time-critical contexts and to initiate further research in this domain. Future work can further explore and contribute to the refinement of these HI-viz guidelines.

### Future Work

Future work could examine the uses and value of these guidelines in different contexts and time-critical domains. For example, in the health care setting, HI-viz can be used to communicate large-scale scientific datasets for quick analysis and assist in collaborative or real-time informed decision-making by providing comprehensive and easily interpretable visual representation of complex patient data, consequently improving patient outcomes.

In a time-critical health care setting such as accident and emergency or intensive care units, HI-viz can be used to provide real-time visualizations of patient data, such as vital signs, symptoms, laboratory test results, medical history, medication or antibiotic administration [2], and triage scores. Where the fast-paced environment of an emergency room requires rapid assessment and prioritization of patients based on the severity of their condition, HI-viz can assist emergency staff in the quick identification and assessment of critical patients and prioritize their care based on the urgency and complexity of their needs as well as facilitating timely, informed decisions for appropriate interventions.

In primary care, general practitioners have highlighted a need to assimilate and understand the patient’s key information within 30 seconds before a consultation [1]. There is a need to prioritize and present key information, including problem history, active and significant past, long-term conditions, medication, demographics (name, age, gender, and other socioeconomic details), and patient stratification, and present this efficiently to enhance situational awareness; enable informed decisions; and, consequently, improve patient health outcomes [1,147-150]. We aim to use the proposed guidelines in an iterative process to design and evaluate a holistic integrated visualization for supporting general practitioners in gaining a quick overview of the patient’s health status before the clinical consultation as they have very limited time before and during clinical consultations [1,147,148,151,152]. The needs and priorities of stakeholders will be engaged through participatory methods (ie, focus groups, workshops, and semi-structured surveys) to ensure the acceptability of the visualization by its users. The effectiveness of the guidelines can be ascertained by how effectively the design intent is achieved (ie, enhancing efficiency, task time for retrieving information, and usability and user experience).

### Conclusions

We argue that HI-viz in time-critical domains is a unique use case requiring a unique set of design guidelines. We propose 5 main guidelines derived from existing design theories and guidelines. We hope that these design guidelines will serve as a starting point to enable both holistic and rapid processing of information, facilitating better-informed decisions in time-critical contexts. As the amount of information available from multiple and heterogeneous sources of data is increasing rapidly, we see significant value in further developing and refining design guidelines for data visualization.

## Appendix

Multimedia Appendix 1 PRISMA (Preferred Reporting Items for Systematic Reviews and Meta-Analyses) checklist.

Multimedia Appendix 2 Features of the holistic, integrated, time, and general guideline characteristics of the studies in the review.

## Abbreviations

HI-viz: holistic integrated information visualization
PRISMA: Preferred Reporting Items for Systematic Reviews and Meta-Analyses

## Data Availability

The datasets generated during and analyzed during this study are available from the corresponding author on reasonable request.
