# Supplementary material for: Toward Guidelines for Designing Holistic Integrated Information Visualizations for Time-Critical Contexts: Systematic Review
Source: J Med Internet Res. 2024 Nov 20;26:e58088. doi: 10.2196/58088 (PMC11618013; doi:10.2196/58088)
Supplement: Multimedia Appendix 2 [file jmir_v26i1e58088_app2.pdf]

Multimedia Appendix 2. Features of the Holistic, Integrated, Time & General Guideline Characteristics of the Studies in the Review

	Holistic						Integrated			Time						General Design								
**Study [reference]**	**Prioritisation**	**Interaction**	**Visual Aggregates**	**Organisation**	**Reduction**	**Abstraction**	**Gestalt Principles**	**Visual Aggregates**	**Exploratory Interaction**	**Pre-attentive Attributes**	**Gestalt Principles**	**Glanceable**	**Abstraction**	**Interaction**	**Task-complexity**	**Reduction**	**Visual Clarity**	**Organisation**	**UX / System Principles**	**Interaction**	**Navigation**	**Relationships**	**Task-complexity**	**Alarms**
Adnan et al. (2008) [98]	-	x	-	-	-	-	-	-	-	-	-	-	-	-	-	-	-	-	-	x	-	-	-	-
Ajani et al. (2022) [99]	-	-	-	-	-	-	-	-	-	-	-	-	-	-	-	x	-	-	-	-	-	-	-	-
Andrews et al. (2011) [100]	-	x	-	-	-	-	-	x	-	-	-	-	-	-	-	-	-	-	-	-	x	x	-	x
Bateman et al. (2010) [101]	-	-	-	-	-	-	-	-	-	-	-	-	x	-	-	x	-	-	-	-	-	-	-	-
Brath, R. (1997) [102]	-	x	-	-	-	-	-	-	-	-	-	-	-	-	-	-	-	-	-	x	-	-	-	-
Chang et al. (2006) [103]	-	-	-	-	-	-	x	-	-	-	-	-	-	-	-	-	-	x	-	-	-	x	-	-
Cowan, N. (2015) [104]	-	-	-	x	-	-	-	-	-	-	-	-	-	-	-	-	x	x	x	-	-	-	-	-
De Carlo et al. (2022) [105]	-	x	-	-	-	-	-	-	-	-	-	-	-	-	-	-	-	-	-	x	-	-	-	-
De Weck et al. (2011) [106]	-	-	-	-	-	-	-	-	-	-	-	-	-	-	-	x	x	-	-	-	-	-	-	-
Dix, A. (2013) [107]	-	x	-	-	-	-	-	-	-	-	-	-	-	-	-	x	x	-	-	-	-	-	-	-
Dybala et al. (2020) [108]	-	-	-	-	-	-	x	-	-	-	x	-	-	-	-	-	-	-	-	-	-	-	-	-
Ellis & Dix (2007) [109]	-	-	-	-	-	-	-	-	-	-	-	-	-	-	-	x	-	-	-	-	-	-	-	-
Elmqvist & Fekete (2010) [9]	-	x	x	-	-	-	-	x	-	-	-	-	-	-	-	-	-	-	-	-	-	-	-	-
Endsley, M.R. (2012) [19]	x	-	-	x	-	-	-	-	-	-	-	-	-	-	-	x	x	x	-	-	-	-	x	x
Fekete & Plaisant (2002) [110]	-	x	-	-	-	-	-	-	-	x	-	-	-	-	-	-	-	-	-	-	-	-	-	-
Forsell & Johansson (2010) [69]	-	-	-	-	-	-	-	-	-	-	-	-	-	-	x	x	x	x	-	-	x	x	x	-
Healey et al. (1995) [111]	-	-	-	-	-	-	-	-	-	x	-	-	-	-	-	-	-	-	-	-	-	-	-	-
Hick, W.E. (1952) [128]	-	-	-	-	-	-	-	-	-	-	-	-	-	-	x	-	-	-	x	-	-	-	-	-
Idrissov et al. (2020) [112]	-	-	-	-	-	-	x	-	-	-	-	-	-	-	-	-	-	-	-	-	-	x	-	-
Kelleher & Wagener (2011) [113]	-	-	-	-	-	-	-	x	-	-	-	-	-	-	-	-	x	-	-	-	-	x	-	-
Kornhauser et al. (2009) [114]	-	-	-	-	-	-	x	-	-	x	-	-	-	-	-	-	x	-	-	-	-	-	-	-
Krekhov et al. (2019) [115]	-	-	-	-	-	-	-	-	-	x	-	-	-	-	-	-	-	-	-	-	-	-	-	-
Ku et al. (2012) [116]	-	x	-	-	-	-	-	-	-	-	-	-	-	-	-	-	-	-	-	-	-	-	-	-
Lima, M. (2019) [5]	-	-	-	-	-	-	-	-	-	-	-	-	-	x	-	-	-	x	-	x	x	-	-	-
Matthews et al. (2006) [17]	-	-	-	-	-	x	-	-	-	-	-	x	x	-	-	-	-	x	-	-	-	-	-	-
Midway, S.R. (2020) [67]	x	-	-	-	-	-	-	-	-	-	-	-	-	-	-	-	x	-	-	-	-	-	-	-
Miller, G.A. (1956) [129]	-	-	-	x	-	-	-	-	-	-	-	-	-	-	-	-	x	x	x	-	-	-	-	-
Moran, K. (2016) [134]	-	-	-	x	-	-	-	-	-	-	-	-	-	-	-	-	x	x	-	-	-	-	-	-
Moreira et al. (2020) [117]	-	-	-	-	-	-	x	-	-	x	x	-	-	-	-	-	-	-	-	-	-	-	-	-
Murray et al. (2017) [118]	-	-	-	-	-	-	-	-	-	-	-	-	-	-	-	-	-	-	-	-	-	x	-	-
Perer & Shneiderman (2009) [119]	-	x	-	-	-	-	-	-	x	-	-	-	-	-	-	-	-	-	-	-	-	-	-	-
Rechtin, E. (1991) [120]	-	-	-	x	-	-	-	-	-	-	-	-	-	-	-	-	-	-	x	-	-	x	-	-
Rodrigues et al. (2006) [121]	-	x	-	-	-	-	-	-	-	x	-	-	-	-	-	-	-	-	-	-	-	-	-	-
Rosli et al. (2015) [122]	-	-	-	-	-	-	x	-	-	-	-	-	-	-	-	-	-	-	-	-	-	-	-	-
Seong & Nuamah (2020) [123]	-	-	-	-	-	-	x	-	-	x	x	-	-	-	-	-	-	-	-	-	-	-	-	-
Shaalan & Jusoh (2020) [124]	-	-	-	-	-	-	-	-	-	-	-	-	-	-	-	-	-	-	x	-	-	-	-	-
Shneiderman, B. (1996) [71]	-	x	-	-	-	-	-	-	-	-	-	-	-	-	-	-	-	-	-	x	-	-	-	-
Spence, R. (2001) [130]	-	x	-	-	-	-	-	-	-	x	-	-	-	-	-	-	-	-	-	x	-	x	-	-
Triesman, A. (1985) [131]	-	-	-	-	-	-	-	-	-	x	-	-	-	-	-	-	-	-	-	-	-	-	-	-
Tufte, E.R. (1983) [132]	-	-	-	-	-	-	-	-	-	-	-	-	-	-	-	x	x	-	-	-	-	-	-	-

van Ham & Perer (2009) [125]	-	x	-	-	-	-	-	-	-	-	-	-	-	-	-	-	-	-	-	-	-	-	-	-
Wagemans et al. (2012) [126]	-	-	-	-	-	-	x	-	-	-	-	-	-	-	-	-	-	-	-	-	-	-	-	-
Wang Baldonado et al. (2000) [90]	-	x	-	-	-	-	-	-	-	-	-	-	-	-	-	-	-	-	-	-	-	-	-	-
Ware, C. (2020) [10]	-	-	-	-	-	-	x	-	-	x	x	-	-	x	-	-	-	-	-	-	-	-	-	-
Wenzel et al. (2003) [127]	-	-	-	-	-	-	-	-	-	-	-	-	-	-	-	-	-	-	-	x	-	-	-	-
Yablonski, J. (2020) [133]	-	-	-	-	x	-	-	-	-	-	-	-	-	-	-	-	-	-	x	-	-	-	-	-
**Total: N= 46 studies**	2	14	1	5	1	1	9	3	1	10	4	1	2	2	2	8	10	8	6	7	3	8	2	2
			**n=22**					**n=13**				**n=16**								**n=30**				
